# Exogenous Nitric Oxide Alleviates Water Deficit and Increases the Seed Production of an Endemic Amazonian Canga Grass

**DOI:** 10.3390/ijms242316676

**Published:** 2023-11-23

**Authors:** Daniela Boanares, Cristiane J. Da-Silva, Keila Jamille Alves Costa, Joana Patrícia Pantoja Serrão Filgueira, Marina Ludmila Oliveira Conor Salles, Luiz Palhares Neto, Markus Gastauer, Rafael Valadares, Priscila Sanjuan Medeiros, Silvio Junio Ramos, Cecilio Frois Caldeira

**Affiliations:** 1 Instituto Tecnológico Vale, Belém 66055-090, PA, Brazil; danielaboanares@gmail.com (D.B.); keila.costa@pq.itv.org (K.J.A.C.); joana.filgueira@pq.itv.org (J.P.P.S.F.); marina.salles@pq.itv.org (M.L.O.C.S.); markus.gastauer@itv.org (M.G.); rafael.borges.valadares@itv.org (R.V.); priscila.sanjuan.medeiros@gmail.com (P.S.M.); silvio.ramos@itv.org (S.J.R.); 2 Department of Horticulture Science, North Carolina State University, Raleigh, NC 27695-7609, USA; cjdasilv@ncsu.edu; 3 Department of Biology, Universidade Estadual do Sudoeste da Bahia, Jequié 45083-900, BA, Brazil; netopalhares1@gmail.com

**Keywords:** Brazilian OCBIL, mining, Poaceae, *Sporobolus multiramosus*, water deficit

## Abstract

Open pit mining can cause loss in different ecosystems, including damage to habitats of rare and endemic species. Understanding the biology of these species is fundamental for their conservation, and to assist in decision-making. *Sporobolus multiramosus* is an annual grass endemic to the Amazon canga ecosystems, which comprise rocky outcrop vegetation covering one of the world’s largest iron ore reserves. Here, we evaluated whether nitric oxide aids *S. multiramosus* in coping with water shortages and examined the physiological processes behind these adaptations. nitric oxide application improved the water status, photosynthetic efficiency, biomass production, and seed production and germination of *S. multiramosus* under water deficit conditions. These enhancements were accompanied by adjustments in leaf and root anatomy, including changes in stomata density and size and root endodermis thickness and vascular cylinder diameter. Proteomic analysis revealed that nitric oxide promoted the activation of several proteins involved in the response to environmental stress and flower and fruit development. Overall, the results suggest that exogenous nitric oxide has the potential to enhance the growth and productivity of *S. multiramosus*. Enhancements in seed productivity have significant implications for conservation initiatives and can be applied to seed production areas, particularly for the restoration of native ecosystems.

## 1. Introduction

Considered a Brazilian old, climatically buffered, infertile landscape (OCBIL), the campos rupestres are characterized as grassy–shrubby vegetation with a mosaic of fire-prone vegetation, including rocky outcrops of quartzite, sandstone, or ironstone (known locally as *canga*) [1]. The cangas are mainly concentrated in the 7200 km^2^ south of Serra do Espinhaço in southeastern Brazil [2] and in a particularly important and extensive canga region located within the Amazon rainforest in the Serra dos Carajás, eastern Amazon [3,4]. Due to the high concentration of iron in the soil, this unique and severe edaphic and climatic conditions impose stress to the plants, leading to adaptive pressure on the vegetation that promotes a specialized flora with a high degree of endemism and different adaptations [5,6,7].

The edaphic peculiarity of canga is associated with the activity of ore extraction. Severe ecosystem degradation by human activities, such as open pit mining, can pose a number of challenges to ecosystem restoration. Due to the great economic and social importance of mining [8], it is inevitable that new mines cause changes in the environment, harming several species, among them rare and endemic ones. For this reason, knowledge of the biology, such as occurrence/distribution, diversity, propagation, and adaptation potential of these species becomes a research priority, as it allows efficient decision-making for their conservation [9].

Changes in environments due to mining activities affect rare and endemic species. *Sporobolus multiramosus* is an example of an annual grass species endemic to Carajás’ canga ecosystems in eastern Amazon/Brazil [10]. This species is classified as a range-restricted endemic, emphasizing the need for further studies to ensure its conservation [7]. In addition to being an endemic species of the region, *S. multiramosus*, which belongs to the family Poaceae, presents interesting characteristics for revegetation or to start land rehabilitation [11]. Grass species can grow rapidly, slowing the erosion process from the ground cover due to dense superficial root strata, helping to keep the substrate more aggregated, in addition to soil cover by leaves reducing the impact of runoff from rainfall [12,13]. However, due to the lack of information about the physiology of the species, the management of native grasses is still quite difficult [13]. In addition, a limiting factor for the use of native species in the recovery of degraded areas is the availability of seeds [14].

The presence of shallow soil and high solar incidence in the canga cause a high evaporative demand, mainly in the dry season, when plants can suffer water stress. In grasses, for example, drought is one of the main factors that impact the production of biomass and seeds [15]. Furthermore, plants exhibit multiple interconnected responses to water stress. Stomatal closure is one such response that allows plants to reduce transpiration but also restricts CO_2_ uptake, leading to a decline in photosynthesis. Consequently, these effects have significant implications for carbon metabolism and the flow of resources between source and sink organs such as seeds, flowers, and fruits [16]. Understanding the physiology of the target species, in addition to assisting in the production of seeds for use in restoration programs, is necessary.

Nitric oxide (NO) is a redox-active, low molecular weight molecule with an important role in plant development [17]. NO is involved in promoting seed germination, photomorphogenesis, mitochondrial activity, leaf expansion, growth, stomatal closure, fruit maturation, senescence, and iron metabolism [18,19,20,21]. In addition to its role in plant development, NO is related to plant tolerance to biotic and abiotic stresses [22,23], aiding in the accumulation of osmolytes, improving the antioxidant system [24], photosynthesis, and biomass accumulation [6]. Among exogenous NO donors, such as S-nitrosoglutathione (GSNO), S-nitroso-N-acetylpenicillamine (SNAP), and sodium diethylamine NONOate (DEA-NONOate), sodium nitroprusside (SNP) is the most widely used to understand NO-mediated responses in plants under water stress [25,26]. SNP is an exogenous plant bioactive signaling molecule with multiple functions, ranging from seed germination to crop growth and production [27,28,29]. It has several applications, such as a seed preparation agent [30], fertigation [31], or foliar spraying [32]. Foliar application has been considered the most effective practice to regulate physiological processes, maintaining water status, and activating antioxidant machinery in plants experiencing water stress [33].

Some studies related to NO and water deficit in Poaceae have focused only on cultivated species, such as maize (*Zea mays*) and rice (*Oryza sativa*), for example [34,35]. SNP influences sulfur and nitrogen assimilation pathways in a dose-dependent manner to improve drought tolerance in maize [36]. In rice, the application of SNP alleviated the effects of water deficit [35]. Considering these findings in these Poaceae species, it is plausible that a native species from the same family may exhibit similar responses to the exogenous application of NO on its leaves.

Therefore, the aim of this study was to investigate the effect of exogenous NO in alleviating water stress in *S. multiramosus*. For this purpose, the plants were grown in a greenhouse under water deficit conditions with application of SNP. Biomass production, water status, photosynthesis, root and leaf anatomy, and protein profiling analysis were performed.

## 2. Results

### 2.1. Relative Water Content and Photosynthetic Efficiency

**Figure 1:**
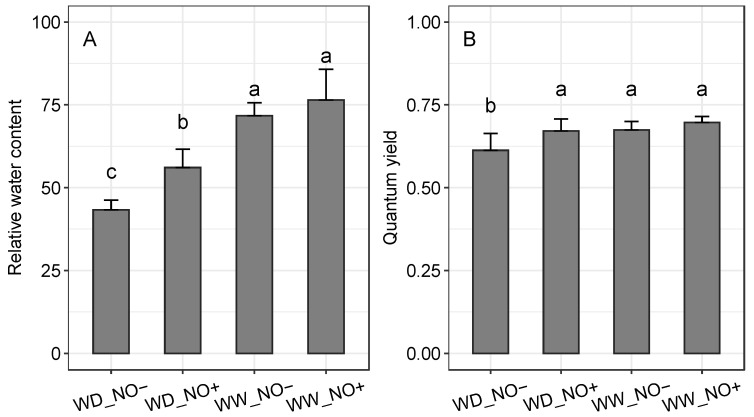
Relative water content (**A**) and quantum yield–QY (**B**) of *Sporobolus multiramosus* plants under different water regimes and NO applications. WD_NO−: water deficit without exogenous NO application; WD_NO+: water deficit with exogenous NO application; WW_NO−: well-watered without exogenous NO application; WW_NO+: well-watered with exogenous NO application. Bars with the same letters indicate no significant pairwise differences between the treatments and control according to a contrast test at 95% probability.

Plants cultivated in well-watered (WW) conditions showed the highest values of relative water content (RWC), regardless NO application. Under water deficit (WD) conditions, NO application was able to improve the plant water status by increasing the RWC from 43% (without NO application) to 56% (Figure 1A). Similarly, the quantum yield (QY) of such plants achieved higher values and showed no significant differences from those of plants cultivated in WW conditions (Figure 1B).

### 2.2. Biomass

**Figure 2:**
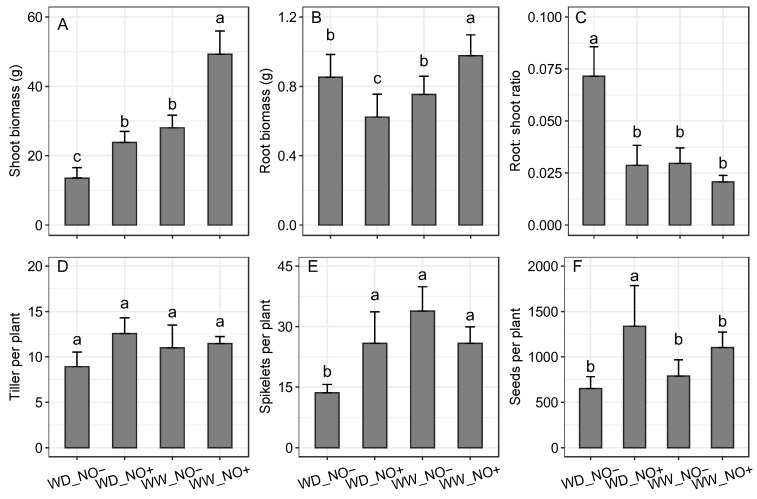
Biomass and reproductive traits of *Sporobolus multiramosus* plants under different water regimes and NO applications: (**A**) shoot biomass, (**B**) root biomass, (**C**) root:shoot ratio, (**D**) number of tillers per plant, (**E**) number of spikelets per plant, (**F**) number of seeds per plant. WD_NO−: water deficit without exogenous NO application; WD_NO+: water deficit with exogenous NO application; WW_NO−: well-watered without exogenous NO application; WW_NO+: well-watered with exogenous NO application. Bars with the same letters indicate no significant pairwise differences between the treatments and control according to a contrast test at 95% probability.

Increments in shoot biomass were observed in plants treated with NO, regardless of water availability (Figure 2A). The lowest shoot biomass was observed in plants under WD that did not receive exogenous NO application. On the other hand, root biomass was lower in plants growing under water deficit that received NO and higher in the WW treatment with NO. Treatments without NO showed intermediate values, regardless of water availability (Figure 2B). The plants cultivated in WD conditions without NO showed the highest values of shoot/root in relation to the other treatments (Figure 2C). There was no difference in the number of tillers among treatments (Figure 2D). In general, the number of spikelets in WD plants was lower than in WW plants (Figure 2E). However, the addition of NO in the WD plants increased the number of seeds per plant (Figure 2F).

### 2.3. Anatomical Traits

**Figure 3:**
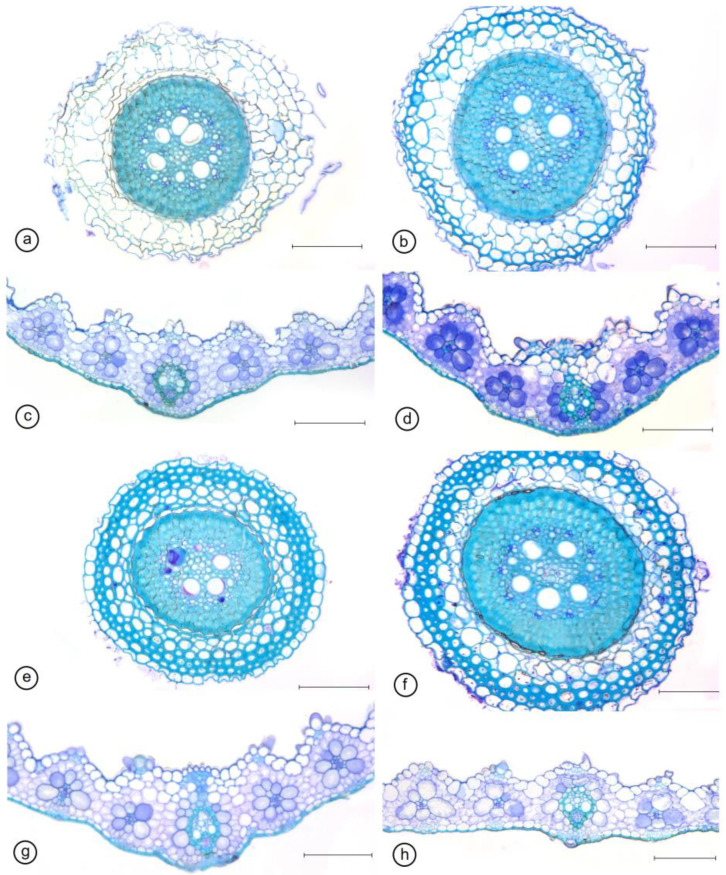
Root and midrib cross sections of *Sporobolus multiramosus* plants under different water regimes and NO applications. Root (**a**,**b**,**e**,**f**) and leaf cross sections (**c**,**d**,**g**,**h**). WD_NO−: water deficit without exogenous NO application (**a**,**c**); WD_NO+: water deficit with exogenous NO application (**b**,**d**); WW_NO−: well-watered without exogenous NO application (**e**,**g**); WW_NO+: well-watered with exogenous NO application (**f**,**h**). Bars: 100 μm

Most of the traits associated with leaf tissue thickness decreased in WD plants compared to WW plants, and the NO application did not affect such traits (Table 1). Only midrib thickness showed a significant reduction in WD plants receiving NO. Density and size of the stomata showed significant differences in relation to NO application both in the WD and WW treatments. In WD plants, the NO application increased the stomatal density and reduced stomata size on the adaxial side of the epidermis, thereby increasing stomatal functionality. In the abaxial epidermis, we observed an increase in stomatal density and in the polar axis and a decrease in the equatorial axis (Table 2, Figure 3). Regarding root anatomy, we observed an overall reduction in epidermis thickness in WD plants, regardless of NO application (Table 3). Also, the root endodermis thickness decreased in the presence of NO, and the WD plants had the lowest values. Similar results were observed for vascular cylinder diameter (Table 3, Figure 3).

### 2.4. Seed Germination

**Figure 4:**
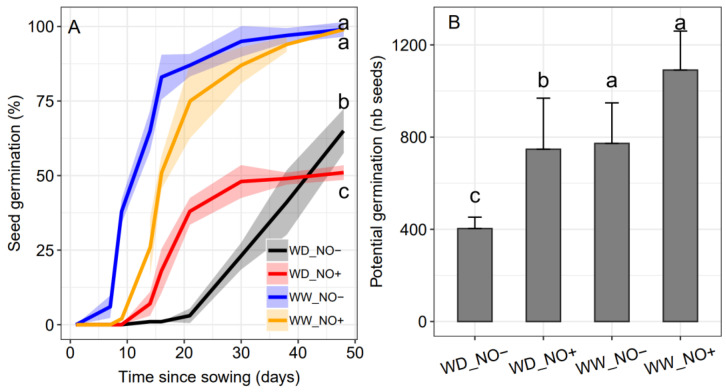
Seed germination rate of *Sporobolus multiramosus* plants under different water regimes and NO applications. (**A**) Percentage of seed germination during 48 days of evaluation and (**B**) number of potential germinating seeds (viable seeds) per plant. WD_NO−: water deficit without exogenous NO application; WD_NO+: water deficit with exogenous NO application; WW_NO−: well-watered without exogenous NO application; WW_NO+: well-watered with exogenous NO application. Lines in panel (**A**) and bars in panel (**B**) with the same letters indicate no significant pairwise difference between the treatments and control according to a contrast test at 95% probability.

The highest rate of seed germination and germination speed index were obtained from plants grown in WW conditions, regardless of NO application (Figure 4A). In WD plants, NO application reduced the time to the start of seed germination (and increased the speed of germination), but reduced the germination rate. Despite this reduction, the NO application in WD plants resulted in a higher number of viable seeds, calculated from the total seeds harvested and germination rate (Figure 4B). When compared to WD plants not receiving NO, we estimate about 33% more potential germinating seeds. In WW conditions, no significant differences were obtained from NO application.

### 2.5. Proteomics Profile

**Figure 5:**
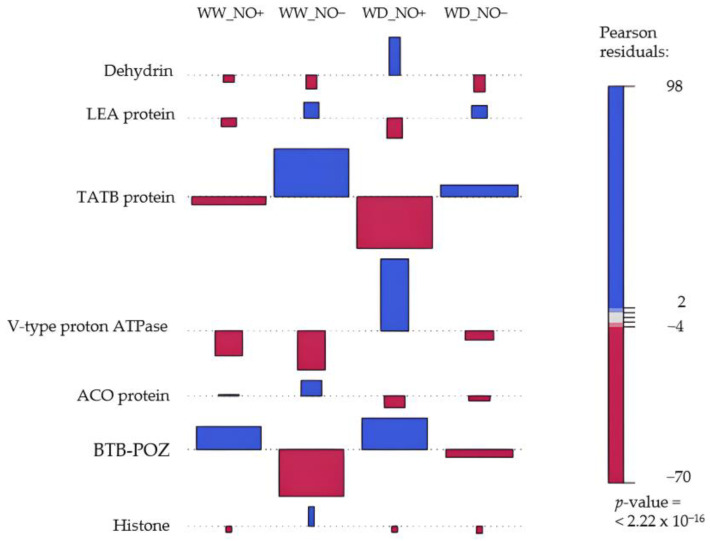
Association between proteins in leaves of *S. multiramosus* under different water regimes and NO applications. Blue columns indicate over representation of proteins, and red columns refer to underrepresented proteins in different treatments. *p* values indicate significance levels for the association between proteins and different treatments. WD_NO−: water deficit without exogenous NO application; WD_NO+: water deficit with exogenous NO application; WW_NO−: well-watered without exogenous NO application; WW_NO+: well-watered with exogenous NO application.

A total of 1949 proteins were identified and quantified from all treatments (Supplemental Information S1). A subset of 127 proteins showed a significant difference (*p* < 0.05) and fold change higher than 1.5 when compared to the control (WW without NO applications). Functional analysis revealed that proteins linked to abiotic stimulus were the most altered within treatments. Among the 45 proteins in this category, we observed that proteins related to water deprivation, namely dehydrins (Q2R4Z7), were 4.44 times more accumulated in WD plants receiving NO applications (Figure 5), while the abundance of Late Embryogenesis Abundant proteins (LEA) (P09441) and sec-Independent Protein Translocase (TATB) (Q9XH75) were 2.64 and 1.64 times reduced, respectively, in WW and WD plants receiving NO. We also observed a significant change in proteins that, in addition to several functions in the plant’s metabolism, are related to the development of flowers and fruits. V-type protein (Q23654) was higher in WD with NO condition. Conversely, the aminocyclopropane carboxylic acid oxidase (ACO) (Q00985) proteins displayed the lowest values in plants of WD with NO application. The NO applications led to the highest amount of BTB/POZ proteins (G8GTN7;C), regardless of water availability. Also, NO applications led to the lowest values of histone acetyltransferase proteins (Q338B9) (Figure 5). In fact, high levels of this protein were observed only in WW without NO applications.

## 3. Discussion

Our findings showed that foliar NO applications significantly improved plant growth attributes of *S. multiramosus* in water deficit. By increasing their RWC, plants accumulated more biomass from improved carbon fixation, as shown by the maintenance of high PSII photosynthetic efficiency and adjustments in root and leaf anatomy and protein profile. Our results agree with previous findings showing the benefits of exogenous NO applications in two turfgrass and Moldavian and balm species under water stress [37,38]. The benefit of NO for plants subjected to water deficit was an increase in shoot biomass and a decrease in root biomass. Due to a probable NO-mediated modulation of ABA and/or auxin signaling in responses to water deficit [6], the higher aboveground biomass for plants under water deficit treated with NO may be a consequence of a change in resource allocation mediated by water stress alleviation. However, the reduction in root mass may have been compensated by the decrease in root diameter, such as the vascular cylinder and endodermis in our study and in *Fagus sylvatica* [39], forming thinner roots more able to absorb water in drought conditions. Thinner roots efficiently enhance root length and surface area, improving the efficiency of carbon invested in roots and greater potential for soil water uptake [40]. It is possible that there was an investment in root hairs, since the relationship between the formation of root hairs in *Arabidopsis thaliana* and exogenous NO was reported both in the initiation phase and in the elongation phase [41], through reorientation of cortical microtubules [42]. Also, decreasing the diameter of the vascular cylinder is a common drought-tolerance response in plants, as it prevents vessel cavitation, a condition that reduces xylem hydraulic conductance [43].

Increments in the root system are commonly reported for plants facing water shortages [44]. As expected, in our results, plants in WD without NO applications altered their carbon allocations to root growth, marked by the increased root:shoot ratio. This change occurred possibly due to adaptive mechanisms inherent to the habitat of *S. multiramosus*, which can often face drought events because of high evaporative demand and low soil water retention capacity in canga ecosystems [5]. Plants in WW conditions treated with NO also showed an increase in root biomass. It has been reported that exogenous NO applications can modulate auxin metabolism, transport, and signaling, raising levels of 3-indoleacetic acid [21] and promoting root growth, in addition to the formation of adventitious roots [41,42,45], corroborating the increment in root biomass observed in WW plants treated with NO in our study. Our results suggest that the impact of exogenous NO on the resource partitioning of the root:shoot ratio may be dependent on the plant’s water status.

The beneficial effects of NO on photosynthesis in *S. multiramosus* under water deficit treatment were found to be similar to those previously described for sugarcane [6,37,46]. One of the reasons that may have caused maintenance in the RWC and photosynthesis in sugarcane and *S. multiramosus* was the increase in the number of stomata and the decrease in their size. Water deficiency generally leads to an increase in stomatal density [34] and a reduction in stomatal size [13]. These changes indicate improved plant adaptation to drought, promoting more sensitive stomatal control and water conservation [47,48]. Small guard cells can cause the stomata to remain open in drought, which demonstrates a balance between carbon gain through photosynthesis and prevention of excessive water loss through transpiration in an adaptive response to the drought condition [49]. Furthermore, the increase in length and decrease in stomatal width under limited water conditions is also an adaptation to water deficit [34].

A greater economy of water and RWC, improvement in photosynthesis, alteration in the allocation of biomass, and root and leaf anatomy change were a consequence of a modification in the production of proteins in plants subjected to water deficit with NO. Plants of have physiologically evolved responses to deal with stress events. Among these responses is the expression of proteins that help plants cope with environmental changes. An example is the dehydrins, which are known as abundant Late Embryogenesis Group II (LEA) proteins [50] that aid in acclimatization/tolerance to water deficit, which were found in greater amounts in plants of *S. multiramosus* submitted to water deficit treated with NO. Dehydrins are one of the most abundant proteins produced in response to drought [51], helping to protect cells against stress [52], changing the RWC and stomatal conductance when plants were exposed to water stress [53]. This corroborates the importance of dehydrin in the maintenance of RWC and quantum yield, and as a consequence, higher carbon assimilation.

On the other hand, in *S. multiramosus*, an increase in the amount of LEA was verified in the WD and WW plants that did not receive exogenous NO. The LEA proteins are macromolecules of various types that are also correlated with tolerance to desiccation, i.e., LEA proteins can reduce water loss during dehydration [54,55]. The fact that LEA increases even in the WW plants may be due to the habitat of this species, which is prone to a lot of heat and high evaporative demand, demonstrating the adaptation of this plant to the environment. *Sporobolus multiramosus* had lower expression of LEA in WD plants treated with NO. Possibly NO promoted tolerance through other pathways, resulting in no need to increase LEA proteins and may have compensated for the higher dehydrin protein production.

The exogenous NO application benefited the seed production of *S. multiramosus* independent of the water regime in which the plants were grown. NO may mediate reversible oxygen balance through its effect on respiratory activity. It is possible that NO also controls energy availability for the synthesis of storage organs such as seed production [56], also justifying the higher seed production in plants in the well-watered treatment. Several factors may have influenced this higher seed production, such as the maintenance of photosynthetic efficiency and RWC, in addition to the high biomass of the aerial parts. Overall, NO application relieved the water deficit and benefited seed production. Metabolically, in addition to the proteins discussed above, related to water deficit, some proteins linked to flower and fruit development, such as vacuolar V-ATPase and BTB/POZ proteins, may have also contributed to the higher seed production in plants receiving exogenous NO.

The increased expression of the BTB/POZ protein may also have influenced the increase in the number of seeds. This protein is involved in promoting the fate and determination of leaves and floral meristems, in addition to promoting the normal growth and development of the stipule and controlling the sizes and/or structures of the inflorescences [57]. The increase in these proteins may have accelerated the phase change, allowing to direct resources from vegetative towards reproductive growth. Among BTB/POZ proteins, the majority interact with cullin (CUL3), indicating their participation in the cullin–RING ligases (CRL3) complex. In addition to their role in embryogenesis, CRL3s also play a crucial role in various developmental processes, including flowering [58]. BOP1 and BOP2, which are members of the BTB-ankyrin family, act as substrate adapters for E3 ligases transmitted through CUL3. Together with the transcription factor LEAFY (LFY) [59], they contribute to the formation of the floral meristem, which could have contributed to the higher number of flowers and consequent increase in seed production observed in plants receiving NO.

The vacuolar V-ATPase is responsible for acidifying a variety of intracellular compartments in eukaryotic cells. In our study, this protein had a significant increase in WD plants treated with NO. It is well known that NO acts as a signaling molecule triggering the activities of this protein [60]. Also, water stress causes excess ROS, which can lead to peroxidation of essential membrane lipids in the plasmalemma or intracellular organelles, resulting in electrolyte leakage of cell membranes. NO plays a protective role in H^+^ pumping activities, membrane integrity, and chlorophyll content [34]. V-ATPase activity is modulated to cope with environmental and metabolic changes [61], and high V-ATPase activity may be associated with a specific role in protecting plants from water stress by maintaining the gradient of protons across the vacuolar membrane. This homeostasis can promote signaling inducing spikelet production, mainly through Ca^2+^, which represents an important signaling molecule and a convergence point of many disparate signaling pathways and necessary for pollen tube growth and elongation [62]. In this way, the greater production of V-ATPase together with BTB/POZ may have positively influenced the seed production in WD plants treated with NO.

Seed production and quality are of interest for conservation, rehabilitation activities, and possible plant cultivation. Many high-quality seeds from an endemic plant facilitate seedling production and their subsequent conservation. The increase in the production of potentially viable seeds from native plants meets the objective of land rehabilitation, especially in mining or steep road slopes, where vegetation faces a significant challenge to establish and thrive. Globally, restoration ecology faces a significant challenge in producing sufficient and high-quality seeds [63]. Therefore, this study highlights that NO has a dual role in plants. It not only helps to relieve water shortage but also contributes to enhancing seed production, particularly in unfavorable circumstances. From our results, it is plausible to consider a possible application to other species. If confirmed, it would be an alternative that could be used even for crops, for which water restriction events have been increasingly frequent and impact grain yield in various parts of the world.

Our results also show that exogenous NO application during the growth period can have substantial changes in the germination of harvested seeds. NO increased the speed of seed germination of WD plants. These data indicate that *S. multiramosus* seeds respond positively to exogenous NO applied during plant development, accelerating germination [19,64]. Internal factors that influence the speed of germination, such as plant hormones like abscisic acid (ABA) and cytokinins, play a crucial role in regulating the germination process. The balance between these hormones can significantly impact the speed and rate of seed germination. Additionally, the energy reserves stored within the seeds, delivered after GA_3_ activation of alpha-amylase, are utilized by the embryo to support initial plant growth. Seeds with higher energy reserves tend to exhibit faster and more vigorous germination [65,66,67]. NO production during the seed germination targets proteins critical for cell elongation (radicle emergence) and growth (coleoptile extension), and NO is known to regulate ion channels in plant cells, increasing Ca^2+^, which affects protein phosphorylation cascades [68]. Producing faster-germinating seeds could be interesting for potential species to be used in the revegetation of degraded areas. In addition to covering soil faster, thereby reducing erosion risks, faster germination can lead to a rapid seedling establishment in the environment and reduce competition with other species and even seed leaching. This characteristic is of interest even for crop species, with the advance in germination and, consequently, in the production of seedlings of agronomic interest. For crop species, it is a great advantage to obtain uniform batches of plants based on germination.

Despite the higher germination speed, a lower germination rate was found in seeds of plants grown in WD conditions treated with NO. A possible reason to the lower germination could be a higher carbon demand than the offer delivered to development of all initiated seeds. It is possible that NO induced greater flowering, but these plants were not able to adequately supply enough nutrients for all forming seeds. Also, the low level of ACO observed in these plants could have interfered in seed germination. ACO also converts the ethylene precursor (ACC) into ethylene. In many cases, ACO controls the production of ethylene during seed germination. Ethylene is implicated in seed formation and germination of many plant species [69,70,71]. This decrease can be related to the fact that NO prevents the production of ethylene and thus senescence [72]. On the other hand, the decrease in ethylene production may interfere with seed maturation. Despite a lower germination rate in WD plants treated with NO, there was a higher seed production that compensates for the low germination. Finally, taken together, our results indicate that we can have a higher number of potential seedlings when compared to plants grown in similar conditions without NO applications, as indicated by the total number of seeds and their germination rate.

## 4. Materials and Methods

### 4.1. Plant Material

Seeds of *Sporobolus multiramosus* were harvested in a field of canga (N1, one of the plateaus of Serra dos Carajás) and germinated in Gerbox^®^ plates containing Germitest^®^ paper in a growth chamber (Fitotron^®^ SGC 120, Weiss Technik, Loughborough, UK) at a temperature of 25 °C and photoperiod of 12:12 h of light:dark. Then, seedlings were transferred to pots of 0.3 L filled with commercial horticultural substrate (Carolina Soil, Santa Cruz do Sul, RS, Brazil). Plants were cultivated in greenhouse conditions.

### 4.2. Experimental Design

After 60 days from potting the seedlings in the substrate “Carolina Soil”, four treatments were applied: plants with water deficit (WD) with or without exogenous NO applications and plants well-watered (WW) with or without NO application. The two water regimes were achieved by maintaining the substrates at around 70% of field capacity (control or WW plants—high soil water availability and plants hydrated, as observed during the rainy periods) and under water shortages with around 20% of field capacity (WD plants—conditions to reduce leaf water content close to 50% of full hydrated plants, similar to dry periods). Field capacity was maintained by weighing the pots before and after irrigations. The NO application started on the same day that irrigation was withdrawn to start the water deficit treatments. Every 15 days for two months, plants were sprayed with a solution containing 100 µM of sodium nitroprusside (SNP, NO donor) diluted in distilled water containing Tween-20 (0.1%). NO applications were carried out during the morning, and the solutions were prepared on the same day of application. In treatments without NO, only water with Tween-20 was applied. Each treatment was composed of 12 pots containing one plant each. After seed maturity, approximately two months after starting foliar NO application, leaf and root samples were collected as described below.

### 4.3. Relative Water Content

Measurements of relative water content (RWC) were obtained from fully developed leaves sampled two days before seed and biomass harvesting. RWC was measured as RWC = 100 × [(FW − DW)/(TW − DW)], where fresh (FW), turgid (TW), and dry (DW) weights of leaf fragments were determined as described by Jamaux et al. (1997) [73]. The FW was measured at leaf sampling and the DW was obtained after drying the samples at 75 °C for at least 24 h.

### 4.4. Photosynthetic Efficiency

To determine the quantum yield (QY), the photosystem II chlorophyll fluorescence was measured using the LI-600 Porometer/Fluorometer (LICOR, Lincoln, NE, USA). All measurements were taken from the middle part of mature leaves exposed to the sun.

### 4.5. Tillering Rate and Biomass Production

The tillering rate and number of spikelets and seeds were determined at the end of the plant growth period. Then, the shoot and root biomass were harvested separately, washed, and dried in an oven at 62 °C until constant weight, using a precision scale (Mettler Toledo, AB265, Greifensee, Switzerland).

### 4.6. Anatomical Analyses

Samples were collected from the middle region of the leaf blades of fully expanded leaves and from the roots at 5 cm from the root apex. Both samples were fixed in FAA (formalin, acetic acid, 70% alcohol) for 24 h [74], and the materials were then isolated, dehydrated in an ethyl series, embedded in methacrylate (Historesin, Leica^®^), and sectioned in a rotary microtome (Leica RM 2245, Leica^®^ Biosystems, Heidelberg, Germany). The cross sections (5 µm thick) were stained with toluidine blue, pH 4.0 [75]. For stomatal characterization, the epidermal impression method was used according to [76]. The samples were observed and photomicrographed under an optical microscope (Zeiss Scope A.1, Zeiss, Wetzlar, Germany) coupled to a digital camera (AxioCam TCc 5, Zeiss, Wetzlar, Germany). The images were analyzed with Image J software (ImageJ 1.x, LOCI, University of Wisconsin, WI, USA), previously calibrated. The adaxial epidermis thickness, abaxial epidermis thickness, bulliform cell thickness, midrib thickness, midrib vascular bundle thickness, bundle sheath thickness, and metaxylem diameter were measured. For both leaf sides, the stomatal density was calculated as the number of stomata per unit area. The root anatomical traits of root epidermis thickness, root endodermis thickness, root cortex diameter, vascular cylinder diameter, and root metaxylem diameter were evaluated.

### 4.7. Germination Assay

The germination assays were carried out with seeds harvested from each treatment. Seeds were sterilized by immersion in a 1% sodium hypochlorite solution for 3 min and washed in sterile water for 1 min. For each treatment, we evaluated five replicates with 20 seeds each, which were placed on Gerbox^®^ plates containing Germitest^®^ paper. This test was carried out in a growth chamber (Fitotron^®^ SGC 120, Weiss Technik, Loughborough, UK) at 25 °C with a photosynthetic photon flux density (PPFD) of 50 µmol m^−2^ s^−1^, photoperiod of 12:12 h day:night, and air relative humidity of 60%. The number of germinated seeds was recorded daily for 48 days, with germination defined as radicle emergence (2 mm). The germination percentage (G%) was calculated as G% = (N/20) × 100, where: N = number of seeds germinated at the end of the test. The germination speed index (GSI) was obtained by GSI = Σ(ni/ti), where: ni = number of seeds that germinated in 30 days; t = time since the start of the test. To determine the total seed germination potential, we multiplied the germination percentage of each treatment by the total number of seeds harvested per plant.

### 4.8. Proteomic Profile

Leaves from *S. multiramosus* were collected, fast frozen in liquid nitrogen, transported to the laboratory, and stored at −80 °C until further use. The leaves were powdered with liquid nitrogen and subjected to protein extraction following the method described by [77] with some modifications. For the extraction of soluble proteins, 3 replicates of 1 g of powdered leaves were weighed and homogenized in 5 mL of SDS buffer (TrisHCl 0.1 M, PMSF 1 mM, and 2% SDS, pH 5.0) with polyvinyl-polypyrrolidone in a ratio of 1:40:2 (*w*/*v*/*w*). The leaf suspension was sonicated on ice prior to the phenol extraction step and ammonium acetate precipitation. Protein pellets were cleaned with ice-cold acetone and ice-cold ethanol and then digested with trypsin.

The identification and quantification of proteins were performed in a nanoACQUITY UPLC^®^ ultra-performance liquid chromatography (Milford, MA, USA), configured for fractionation in two dimensions as reported by Herrera et al. (2018) [78]. Five micrograms of the peptides were analyzed with three analytical replicates. The first dimension used a 5 µm XBridge BEH130 C18 (300 µm × 50 mm) and a Symmetry C18 5 µm (180 µm × 20 mm) trapping column at a flow rate of 2000 nL min^−1^. The second dimension used a 1.7 µm BEH130 C18 1.8 µm (100 µm × 100 mm) analytical column, at a flow rate of 400 nL min^−1^. The samples were separated into five fractions with a gradient of 10.8, 14.0, 16.7, 20.4, and 65.0% acetonitrile. The chromatograph was coupled to a NanoLock ESI-Q-ToF SYNAPT G2-S (Waters Co., Milford, MA, USA) mass spectrometer. The acquisition ranged from 50 to 2000 Da, in MSE mode (data-independent analysis) at a scan rate of 0.5 s and an interscan delay of 0.1 s. The data were processed using the Progenesis QI software (version 3.0) for identification and quantification, using the Viridiplantae database from UniProt (UniProtKB/swissprot, uniprot.org, accessed on 10 July 2022). The functional annotation of proteins was performed using the OmicsBox v2.1.14 (bioBam, Valencia, Spain) and Uniprot (UniProtKB/Swiss-prot, uniprot.org, accessed on 17 August 2022) in the Kyoto Encyclopedia of Genes and Genomes (KEGG). The proteins were analyzed comparatively, evaluating the fold-change between the means of irrigated and non-irrigated treatments, in order to assess whether the plant expressed some type of stress.

### 4.9. Data Analysis

Differences in the biomass, RWC, QY (quantum yield) and leaf and root anatomical traits among the treatments were tested using one-way analysis of variance (ANOVA) followed by a post hoc Tukey HSD test.

The significance levels of the differential abundances of proteins were determined by applying the ANOVA test (*p* < 0.05). To visualize differences in protein profiles between treatments, a nonmetric multidimensional scaling (NMDS) analysis followed by a PERMANOVA (‘vegan’ R package, ‘adonis’ function) was applied. Post hoc tests were performed with the ‘pairwise.adonis’ function. We used a contingency table to verify associations between proteins and different treatments. The results were visualized by means of an association plot produced by the function ‘assoc’ in the R package ‘vcd’ [79]. All analyses were done using the R platform [80], and figures were prepared using the ‘ggplot2’ package.

## 5. Conclusions

Physiological knowledge of native species in the Amazonian canga is becoming increasingly necessary to assist in their conservation. To our knowledge, this is the first time that the use of sodium nitroprusside has been reported in a native Amazonian canga species aiming to improve its tolerance to water deficit and seed production. This study showed that *S. multiramosus* responded positively to the addition of exogenous NO applications, alleviating the water deficit effects by maintaining the RWC and photosynthetic efficiency, consequently leading to greater growth and seed production. NO promoted the activation of several proteins involved in the response to environmental stress, enabling *S. multiramosus* to cope with water deficit challenges. Furthermore, our results also suggest a potential benefit of exogenous NO applications regardless of the water status. These findings are of paramount importance for decision-making on conservation practices for this endemic species. The increase in seed productivity has implications for conservation efforts and areas designated for seed production, such as the restoration of native ecosystems. Additionally, these results encourage further research with other species, particularly grass species promising for mineland rehabilitation, as they may not produce or produce only small quantities of viable seeds.

## Figures and Tables

**Table 1:** The leaf anatomy traits in *Sporobolus multiramosus* submitted to different water regimes and NO applications. WD_NO−: water deficit without exogenous NO application; WD_NO+: water deficit with exogenous NO application; WW_NO−: well-watered without exogenous NO application; WW_NO+: well-watered with exogenous NO application. Mean values and standard deviations (mean ± sd) with the same letters indicate no significant pairwise differences among the treatments and control according to a contrast test at 95% probability.

Substrate	ETAd (µm)	ETAb (µm)	BCT (µm)	MT (µm)	BST (µm)	VBT (µm)	MD (µm)
WD_NO−	11.47 ± 1.2 b	5.38 ± 0.7 b	21.22 ± 2.9 b	126.09 ± 14.9 a	81.34 ± 9.5 a	45.63 ± 5.4 b	13.18 ± 0.81 b
WD_NO+	11.33 ± 0.8 b	5.7 ± 0.7 b	19.45 ± 2.9 b	110.74 ± 5.9 b	84.79 ± 5.9 a	44.32 ± 2.6 b	13.98 ± 0.71 b
WW_NO−	15.15 ± 0.9 a	9.19 ± 1.2 a	27.53 ± 1.6 a	121.83 ±7.8 ab	85.59 ± 6.6 a	50.3 ±3.1 ab	15.74 ± 1.71 a
WW_NO+	15.45 ± 0.9 a	6.69 ± 0.7 b	25.44 ± 1.4 a	127 ± 13.5 a	85.6 ± 6.0 a	52.73 ± 4.3 a	14.65 ± 1.14 a

ETAd: epidermis thickness from adaxial leaf side, ETAb: epidermis thickness from the abaxial leaf side, BCT: bulliform cells thickness, MT: midrib thickness, BST: the bundle sheath thickness, VBT: midrib vascular bundle thickness, MD: metaxylem diameter

**Table 2:** Stomatal traits in *Sporobolus multiramosus* submitted to different water regimes and NO applications. WD_NO−: water deficit without exogenous NO application; WD_NO+: water deficit with exogenous NO application; WW_NO−: well-watered without exogenous NO application; WW_NO+: well-watered with exogenous NO application. Mean values and standard deviations (mean ± sd) with the same letters indicate no significant pairwise differences among the treatments according to a contrast test at 95% probability.

Substrate	SD-Ad (Stomata per mm^2^)	PD-Ad (µm)	ED-Ad (µm)	SF-Ad	SD-Ab (Stomata per mm^2^)	PD-Ab (µm)	ED-Ab (µm)	SF-Ab
WD_NO−	162.3 ± 11.9 b	22.6 ± 1.0 b	12.5 ± 1.3 a	1.83 ± 0.2 b	69 ± 9.6 b	27.3 ± 1.4 b	13.4 ± 1.1 c	2.06 ± 0.2 b
WD_NO+	176 ± 12.5 a	22.2 ± 1.4 b	11.3 ± 1.0 a	1.97 ± 0.2 a	98 ± 11.8 a	29.7 ± 2.1 a	12.2 ± 0.7 d	2.45 ± 0.2 a
WW_NO−	139 ± 14.6 c	24.5 ± 1.2 a	13.9 ± 1.0 a	1.76 ± 0.1 c	61 ± 8.4 b	29.4 ± 2.5 a	16.1 ± 0.9 a	1.84 ± 0.2 c
WW_NO+	131.3 ± 13.9 c	24.7 ± 2.4 a	13.5 ± 1.2 a	1.84 ± 0.2 b	73 ± 14.3 b	25.9 ± 1.9 c	14.5 ± 0.9 b	1.80 ± 0.2 c

SD: stomatal density from the adaxial leaf side, PD: polar diameter, ED: equatorial diameter, SF: stomatal functionality, Ad: adaxial leaf side, Ab: abaxial leaf side.

**Table 3:** Root anatomical traits in *Sporobolus multiramosus* submitted to different water regimes and NO applications. WD_NO−: water deficit without exogenous NO application; WD_NO+: water deficit with exogenous NO application; WW_NO−: well-watered without exogenous NO application; WW_NO+: well-watered with exogenous NO application. Mean values and standard deviations (mean ± sd) with the same letters indicate no significant pairwise differences among the treatments according to a contrast test at 95% probability.

Substrate	RET (µm)	RDT (µm)	RCT (µm)	VCD (µm)	RMD (µm)
WD_NO−	10.5 ± 0.65 c	7.12 ± 0.95 b	70.98 ± 6.96 a	260.55 ± 30.13 a	35.02 ± 2.83 a
WD_NO+	10.5 ± 1.42 c	5.29 ± 1.82 c	79.38 ± 12.13 a	226.4 ± 14.98 b	31.55 ± 5.24 a
WW_NO−	21.37 ± 0.79 b	9.52 ± 1 a	79.27 ± 13.37 a	225.01 ± 31.37 b	31.67 ± 3.47 a
WW_NO+	17.15 ± 1.26 a	7.9 ± 0.59 b	75.72 ± 5.97 a	248.08 ± 38.38 a	30.23 ± 3.84 a

RET = root epidermis thickness, RDT = root endodermis thickness, RCT = root cortex thickness, VCD = vascular cylinder diameter, RMD = root metaxylem diameter.

## Data Availability

The data presented in this study are available in the article and in the Supplementary Materials.

## Supplementary Materials

The following supporting information can be downloaded at: https://www.mdpi.com/article/10.3390/ijms242316676/s1.
